# Higher levels of myelin are associated with higher resistance against tau pathology in Alzheimer’s disease

**DOI:** 10.1186/s13195-022-01074-9

**Published:** 2022-09-24

**Authors:** Anna Rubinski, Nicolai Franzmeier, Anna Dewenter, Ying Luan, Ruben Smith, Olof Strandberg, Rik Ossenkoppele, Martin Dichgans, Oskar Hansson, Michael Ewers, the Alzheimer’s Disease Neuroimaging Initiative (ADNI)

**Affiliations:** 1 Institute for Stroke and Dementia Research, University Hospital, LMU Munich, Munich, Germany; 2 Department of Neurology, Skåne University Hospital, Lund, Sweden; 3 Clinical Memory Research Unit, Department of Clinical Sciences Malmö, Lund University, Lund, Sweden; 4 Department of Neurology, Alzheimer Center Amsterdam, Amsterdam Neuroscience, Vrije Universiteit Amsterdam, Amsterdam UMC, Amsterdam, Netherlands; 5 Munich Cluster for Systems Neurology (SyNergy), Munich, Germany; 6 German Center for Neurodegenerative Diseases (DZNE), Munich, Germany; 7 Memory Clinic, Skåne University Hospital, Malmö, Sweden

**Keywords:** Myelin, Myelin water fraction, Tau-PET, Amyloid-PET, Alzheimer’s disease, Tau spreading, Resistance

## Abstract

**Background:**

In Alzheimer’s disease (AD), fibrillar tau initially occurs locally and progresses preferentially between closely connected regions. However, the underlying sources of regional vulnerability to tau pathology remain unclear. Previous brain-autopsy findings suggest that the myelin levels—which differ substantially between white matter tracts in the brain—are a key modulating factor of region-specific susceptibility to tau deposition. Here, we investigated whether myelination differences between fiber tracts of the human connectome are predictive of the interregional spreading of tau pathology in AD.

**Methods:**

We included two independently recruited samples consisting of amyloid-PET-positive asymptomatic and symptomatic elderly individuals, in whom tau-PET was obtained at baseline (ADNI: *n* = 275; BioFINDER-1: *n* = 102) and longitudinally in a subset (ADNI: *n* = 123, mean FU = 1.53 [0.69–3.95] years; BioFINDER-1: *n* = 39, mean FU = 1.87 [1.21–2.78] years). We constructed MRI templates of the myelin water fraction (MWF) in 200 gray matter ROIs and connecting fiber tracts obtained from adult cognitively normal participants. Using the same 200 ROI brain-parcellation atlas, we obtained tau-PET ROI values from each individual in ADNI and BioFINDER-1. In a spatial regression analysis, we first tested the association between cortical myelin and group-average tau-PET signal in the amyloid-positive and control groups. Secondly, employing a previously established approach of modeling tau-PET spreading based on functional connectivity between ROIs, we estimated in a linear regression analysis, whether the level of fiber-tract myelin modulates the association between functional connectivity and longitudinal tau-PET spreading (i.e., covariance) between ROIs.

**Results:**

We found that higher myelinated cortical regions show lower tau-PET uptake (ADNI: rho =  − 0.267, *p* < 0.001; BioFINDER-1: rho =  − 0.175, *p* = 0.013). Fiber-tract myelin levels modulated the association between functional connectivity and tau-PET spreading, such that at higher levels of fiber-tract myelin, the association between stronger connectivity and higher covariance of tau-PET between the connected ROIs was attenuated (interaction fiber-tract myelin × functional connectivity: ADNI: *β* =  − 0.185, *p* < 0.001; BioFINDER-1: *β* =  − 0.166, *p* < 0.001).

**Conclusion:**

Higher levels of myelin are associated with lower susceptibility of the connected regions to accumulate fibrillar tau. These results enhance our understanding of brain substrates that explain regional variation in tau accumulation and encourage future studies to investigate potential underlying mechanisms.

**Supplementary Information:**

The online version contains supplementary material available at 10.1186/s13195-022-01074-9.

## Background

Alzheimer’s disease (AD) is defined by the presence of beta-amyloid (Aβ) plaques and tau neurofibrillary tangles in the brain. The increase in tau pathology is closely associated with neurodegeneration and symptomatic worsening in AD [1–3], demonstrating a key role of tau in the development of dementia. Accumulation of tau pathology during the course of AD occurs gradually in a highly regional and connectivity-dependent manner, as fibrillar tau pathology typically starts in circumscribed brain areas and subsequently progresses to anatomically connected brain areas [4]. The entorhinal cortex is the prevailing epicenter of early fibrillar tau deposition [5–7], from where pathologic tau subsequently progresses to connected higher cortical areas including the medial frontal and posterior parietal cortex [8–12]. Even at advanced disease stages of globally distributed pathologic tau, some brain regions including the somatosensory and the primary motor cortex remain mostly spared [5]. Together, these findings suggest a highly region-dependent susceptibility to tau-pathology that spreads within a network of closely connected brain regions. However, the biological substrates that underlie the region-specific vulnerability to develop tau pathology remain elusive [13]. Their identification could provide key insights into endogenous factors of resistance against the development of fibrillar tau and may be useful for patient-tailored prediction of disease progression.

Here we investigated whether differences in the level of gray-matter and fiber-tract myelin are associated with lower regional fibrillar tau development. The overall rationale is that ontogenetically lower myelinated brain regions and fiber tracts are more vulnerable, where myelin damage may facilitate the development of tau pathology in AD. Myelin constitutes the lipid-rich insulating membrane that ensheaths the nerve fibers [14]. Heterochronicity of nerve-fiber myelination, which can last until the 4th decade of life [15, 16], entails differences in the level of myelination of major fiber tracts and the connecting gray matter regions: primary sensory and motor cortices are more thickly myelinated, whereas late-maturating higher cortical levels of the temporo-parietal multimodal association cortices are thinly myelinated [16–18]. In AD, lower myelinated fiber tracts are most vulnerable [19–21]. The impairment of myelin may enhance the development of pathologic tau [22], potentially through rendering microglia that are overburdened by lipid uptake in a dysfunctional state [23], and thus, a suboptimal immune response to developing AD pathology [24, 25]. A regional correspondence between regions of higher myelin and lower tau pathology has been noted previously based on visual inspection of brain autopsy studies [26]. However, whether regions of higher myelination are associated with lower levels of regional tau pathology and the progressive spreading between connected brain areas has not been tested so far.

Therefore, our first major aim was to assess whether higher myelinated cortical regions show lower susceptibility to the development of fibrillar tau pathology. To this end, we combined an MRI-derived template of myelin in the brain [27] with cross-sectional PET imaging of fibrillar tau, obtained from deeply phenotyped elderly participants from two independent cohorts: the Alzheimer’s Disease Neuroimaging Initiative (ADNI) [28] and BioFINDER-1 (https://clinicaltrials.gov/ct2/show/NCT01208675).

Our second major aim was to test whether fiber-tract levels of myelin modulated the longitudinal spreading of fibrillar tau in the brain. We previously reported that increases in tau-PET levels are more similar in connected regions showing close functional connectivity [9, 29], supporting the notion that tau spreads preferentially between closely connected brain regions. Here we tested the hypothesis that the level of myelination of the fiber tracts modulates the connectivity-based spreading of tau. Specifically, we hypothesized that higher myelination of fiber tracts is associated with disproportionally lower tau-PET increases in the connected brain regions. In order to address this hypothesis, we combined the myelin maps with MRI-assessed structural and functional connectomes from the human connectome project (HCP) to predict longitudinal tau-PET accumulation over a time period of 1–4 years across both cohorts. Since higher levels of amyloid-PET are a major risk factor of increased cortical tau-PET, we assessed in a sensitivity analysis, whether any associations between myelin and tau-PET can be attributed to amyloid-PET levels. Our study thus investigates in a comprehensive manner in two independent samples myelin as a potentially protective factor against the progression of tau pathology.

## Methods

### Participants

The current study included participants from two large cohort studies: ADNI and the Swedish BioFINDER-1 study. For our main analyses, participants were selected based on the availability of baseline T1-weighted MRI and [^18^F]-AV1451 (flortaucipir) tau-PET. For sensitivity analyses, we also acquired all available [^18^F]-AV45 (Florbetapir) amyloid-PET in ADNI or [^18^F]Flutemetamol amyloid-PET in BioFINDER-1.

For ADNI, the Aβ status (Aβ-/+) was determined based on global amyloid-PET levels, where designation of abnormally elevated amyloid deposition (Aβ +) was defined by the cut-off > 1.11 of global standardized uptake value ratio (SUVR) for [^18^F]AV45-Florbetapir-PET, or a global SUVR > 1.08 for [^18^F]Florbetaben-PET as established previously [30]. Participants were clinically diagnosed as cognitively normal (CN, Mini-Mental State Exam (MMSE) > 24, Clinical Dementia Rating (CDR) = 0, non-depressed), mildly cognitively impaired (MCI, MMSE > 24, CDR = 0.5, objective memory loss on the education adjusted Wechsler Memory Scale II, preserved activities of daily living), or demented (AD, MMSE of 20 to 26, CDR > 0.5, NINCDS/ADRDA criteria for probable AD). We included a group of participants within the AD spectrum consisting of 119 CN Aβ+, 97 MCI Aβ+ , and 59 Aβ+ AD dementia participants. As a control sample we included 199 CN Aβ-/Tau- individuals. Tau negativity was established based on a temporal meta region-of-interest (ROI, including the amygdala, entorhinal cortex, fusiform, parahippocampal, and inferior temporal and middle temporal gyri) cut-off < 1.29, which was shown to discriminate non-AD from AD participants [31]. Ethical approval was obtained by the ADNI investigators at each participating ADNI site, all participants provided written informed consent.

For BioFINDER-1, Aβ status was determined based on global [^18^F]Flutemetamol-PET levels, where abnormally elevated amyloid deposition was defined as a global SUVR cut-off > 0.575 as described previously [32]. The inclusion and exclusion criteria and diagnostic criteria for BioFINDER-1 have been published previously [33]. We included a control sample of 36 CN Aβ-/Tau-, and a group of participants within the AD spectrum consisting of 30 CN Aβ+ , 26 MCI Aβ+ , and 46 Aβ+ AD dementia participants. All BioFINDER-1 participants gave written informed consent to participate in the study prior to inclusion in the study. Ethical approval was given by the ethics committee of Lund University, Sweden. Imaging procedures were approved by the Swedish Medical Product Agency and the Radiation Safety Committee at Skåne University Hospital, Sweden.

### MRI and PET acquisition and preprocessing in ADNI

In ADNI, structural MRI data was acquired on 3T scanning platforms using T1-weighted MPRAGE sequences using unified scanning protocols across sites (image acquisition procedures can be found on: http://adni.loni.usc.edu/methods/mri-tool/mri-analysis/). T1-weighted images and PET scans were taken up to 6 months apart (median interval time = 33 [− 149–176] days).

Tau-PET was assessed with a standardized protocol using 6 × 5 min frames, 75–105 min post-injection of [^18^F]-AV1451. Similarly, amyloid-PET was acquired in 4 × 5 min frames, 50–70 min post-injection of [^18^F]-AV45. The dynamically acquired frames were coregistered and averaged, and further standardized with respect to the orientation, voxel size, and intensity by the ADNI PET core to produce uniform single tau-PET [34].

T1-weighted MRI images and PET data were preprocessed using the Advanced Normalization Tools (ANTs) toolbox (http://stnava.github.io/ANTs/). First, PET images were rigidly co-registered to the participant’s T1-weighted MRI image in native space. Using the ANTs cortical thickness pipeline, T1-weighted images were bias field corrected, brain extracted, and segmented into gray matter, white matter, and cerebrospinal fluid tissue maps. Using ANTs high-dimensional warping algorithm [35] the preprocessed T1-weighted MRI images were further non-linearly normalized to Montreal Neurological Institute (MNI) space. By combining the normalization parameters, we further transformed the Schaefer 200 ROI cortical brain atlas parcellation [36] and the reference regions for intensity normalization of PET images from MNI space to native space. Subsequently, the Schaefer parcellation and the reference regions were masked with subject-specific gray matter masks that were binarized at a probability threshold of 0.3.

**Fig. 1:**
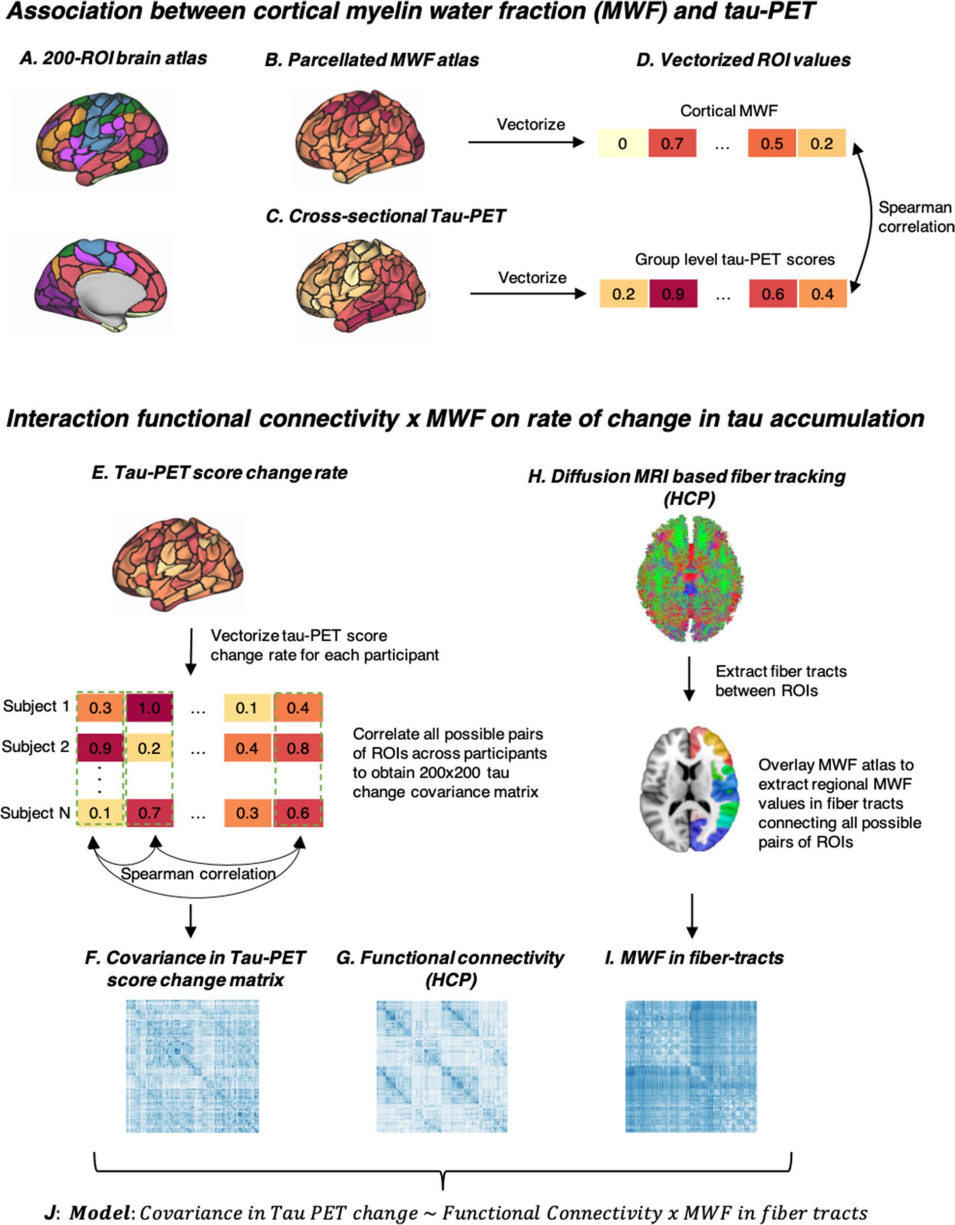
Analysis flow chart. **A** Surface rendering of the 200-ROI brain atlas, based on which we estimated **B** cortical MWF and **C** group-level tau-PET scores, which were further vectorized (**D**) and spatially correlated. **E** The same brain atlas shown in (**A**) was applied to each participant’s tau-PET score change rates, individual values were vectorized and all possible pairs of ROIs across participants were correlated to obtain a covariance in tau-PET score change matrix (**F**). **G** Using the 200 ROI brain atlas shown in (**A**), resting-state fMRI functional connectivity was assessed on 100 participants of the human connectome project (HCP). **H** Diffusion MRI from the HCP was used to estimate the fiber-tracts between each pair of ROIs in the brain atlas shown in (**A**). **I** The MWF atlas was overlayed to extract regional MWF values of underlying fiber tracts. **J** Linear regression analysis was performed with the covariance in tau-PET score change as the dependent variable, and the interaction of functional connectivity by MWF in fiber-tract as the predictor. In a sensitivity analysis, we further controlled the above-mentioned analyses for regional amyloid-PET levels or the covariance in amyloid-PET change (not shown)

PET SUVR images were computed by intensity normalizing PET images to the mean tracer uptake of the inferior cerebellar gray matter for tau-PET data, or to the mean tracer uptake of the whole cerebellum for amyloid-PET data, following previous recommendations [37, 38]. Mean PET SUVR values were extracted for each subject for the 200 cortical ROIs (Fig. 1C).

### MRI and PET acquisition and preprocessing in BioFINDER-1

In BioFINDER-1, T1-weighted MPRAGE (1 mm isotropic, TR/TE = 1900/2.64 ms) and FLAIR images (0.7 × 0.7 × 5 mm^3^, 23 slices, TR/TE = 9000/81 ms) were acquired for all participants on a 3T Siemens Skyra scanner (Siemens Healthineers, Erlangen, Germany).

Tau-PET was acquired 80–100 min after bolus injection of [^18^F]AV1451 on a GE Discovery 690 PET scanner (General Electric Medical Systems, Milwaukee, WI, USA).

The image data was processed by the BioFINDER-1 imaging core using a previously described pipeline developed at Lund University [39]. In brief, MRI images were skull stripped using the combined MPRAGE and FLAIR data, segmented into gray and white matter, and non-linearly normalized to MNI space. PET images were attenuation corrected, motion corrected, summed, and coregistered to the MRI images.

In line with the ADNI data, SUVR images were computed by using the inferior cerebellar gray matter as a reference region, and mean tau-PET SUVR values were extracted for each subject for the 200 cortical ROIs.

### Transforming tau-PET SUVRs to tau-PET scores

The AV1451 tau-PET tracer shows off-target binding which can confound the measurement of fibrillar tau. In order to enhance the specificity of the tau-PET tracer to assess fibrillar tau, we employed gaussian mixture modeling to tau-PET data to separate the target signal from the background signal as previously described [7, 12]. The underlying rationale is that the variability of regional tau-PET values arises from two sources including off-label bindings observed mostly in regions free of fibrillar tau and those with abnormally increased fibrillar tau (such as present in Aβ + participants), resulting in a bimodal distribution of ROI values centered around a mean of off-label tracer binding and a distribution of ROI values centered around a mean of on-target tracer binding. For a given participant’s ROI value, a tau-positivity probability score (i.e. the probability that the tau-PET ROI value reflects abnormal fibrillar tau) can be calculated based on the percentiles of the off-label and target ROI value distributions. In the current study, we applied gaussian mixture modeling to 200-ROI tau-PET SUVRs across participants separately in each study including ADNI and BioFINDER-1. We computed for each participant and ROI the probability to belong either to the off-target or on-target distribution as described previously by us [12]. The resulting tau-positivity probabilities were then multiplied by the original tau-PET SUVRs in order to obtain tau-positivity weighted tau-PET scores.

### Assessment of covariance in tau change

Using longitudinal tau-PET data, we then computed the annual tau change within each of the 200 ROIs for each participant. This was done by computing for each ROI the baseline vs follow-up differences in tau-PET scores divided by the time (in years) between the tau scans. Covariance in tau change (the correlation between tau change levels between pair of regions) was computed by correlating regional tau-PET score change levels between each pair of ROIs across participants using Spearman’s correlation. Resulting in a single 200 × 200 sized covariance in tau change matrix for both cohorts. We set autocorrelations to zero and further Fisher-*z*-transformed all correlations. Similarly, for sensitivity analyses, we computed the covariance in amyloid-PET change as well as a covariance in tau change, using Spearman's partial correlation controlled for age, sex, education, APOE ε4 status, diagnosis, and site.

### Assessment of cortical and fiber tract myelin water fraction (MWF)

Cortical and fiber tracts myelination was determined based on a normative MWF atlas derived from myelin water imaging of healthy individuals (*n* = 50, mean age 25 years) [27]. To determine the regional cortical distribution, we applied a neocortical 200 ROI brain parcellation (Fig. 1A; 36) to the myelin water atlas and extracted the regional mean MWF within each of the 200 ROIs (Fig. 1B). To determine the myelin content within the fiber-tracts connecting each pair of ROIs, first, we obtained minimally preprocessed diffusion-weighted images (DWI) [40] from 100 participants of the human connectome project (HCP). We further used a multi-shell multi-tissue constrained spherical deconvolution and probabilistic tractography pipeline, as previously described [12]. Using the same 200 ROI parcellation, we defined nodes and assigned for each pair of ROIs the reconstructed fiber tract streamlines (Fig. 1H). The myelin water atlas was then applied to the reconstructed streamlines, and the mean myelin value along the streamlines between each of the two regions was extracted. This resulted in a 200 × 200 matrix of the mean MWF in fiber tracts between each pair of ROIs. From the resulting 100 MWF matrices, we computed the group-average MWF in fiber tracts matrix (see Fig. 1I).

### Assessment of functional connectivity

To determine a functional connectivity template, we downloaded spatially normalized, minimally preprocessed resting-state fMRI images from the same 100 participants of the HCP. We then applied detrending, band-pass filtering (0.01–0.08 Hz), despiking, and motion correction to the HCP resting-state data. For each individual, we assessed the functional connectivity values by Fisher-*z*-transformed Pearson-moment correlations between all possible ROI pairs in the Schaefer parcellation. From the resulting 100 functional connectivity matrices, we computed the group-average functional connectivity matrix (Fig. 1G).

### Statistics

Within each cohort, subject characteristics were compared between groups using Kruskal–Wallis for continuous measures (post hoc Dunn’s tests adjusted for multiple comparisons) or Chi-squared (*χ*^2^) tests for categorical measures.

In order to test the association between cortical MWF and group-averaged tau-PET scores in corresponding gray matter ROIs, we computed a spatial correlation using Spearman’s rank correlation (Fig. 1B–D) separately for ADNI and BioFINDER-1. As sensitivity analyses, we first repeated this analysis using tau-PET SUVRs as the dependent variable, and secondly we tested whether the association differed by APOE ε4 status (ε4 allele carriers vs non-carriers). To this end, we first stratified participants by APOE ε4 status and determined the bootstrapped distribution of the correlation coefficients (rho) between MWF and tau-PET scores for each of the two APOE groups. Next, we randomly sampled from the participants pool with replacement in each of the APOE groups, and we computed the 95% confidence intervals (CI) of the rho-value distribution within each of the groups, using 1000 bootstrapping iterations.

Next, we assessed whether the myelin levels of the fiber tracts are a modulating factor of connectivity-based spreading of tau. To that end, using linear regression we tested the interaction functional connectivity by MWF in fiber-tracts on the covariance in tau-PET score change separately for ADNI and BioFINDER-1. In additional analyses, we controlled the assessment of covariance in tau change for age, sex, APOE ε4 status, diagnosis, and site.

Finally, in order to assess whether the observed associations in the current study are driven by regional amyloid pathology, we repeated the above-mentioned analyses controlling for baseline amyloid-PET levels or covariance in amyloid-PET change based on the analysis.

All statistical analyses were performed using R statistical software. Brain surface renderings were created in a connectome workbench. All effects were considered significant when meeting an α-threshold of 0.05.

## Results

We included a total of 612 participants consisting of non-demented and demented individuals with biomarker evidence of AD including elevated amyloid-PET accumulation (Aβ + , ADNI: *n* = 275; BioFINDER-1: *n* = 102) and CN controls without elevated amyloid-PET and tau-PET (Aβ-/Tau-, ADNI: *n* = 199; BioFINDER-1: *n* = 36, see Table 1).

**Table 1:** Sample demographics, mean (SD)

**ADNI **					
	**CN Ab-/Tau-** **(*n*=199)**	**CN Ab+** **(*n*=119)**	**MCI Ab+** **(*n*=97)**	**AD dementia** **(*n*=59)**	***p*-value**
Age, years	70.88 (6.40)^†,‡,§^	74.64 (7.39)^*^	74.77 (7.40)^*^	76.20 (9.33)^*^	<0.001
Sex, M/F	72 / 127	44 / 75	51 / 46	31 / 28	0.011
Education, years	16.78 (2.28)^§^	16.68 (2.38)^§^	16.11 (2.46)	15.44 (2.41)^*,†^	<0.001
MMSE	29.19 (1.07)^‡,§^	28.91 (1.36)^‡,^^§^	27.49 (2.19)^*,†,^^§^	22.19 (3.96)^*,†,‡^	<0.001
APOE e4 carriers -/+^a^	136- / 51+^†,‡,§^	54- / 60+^*^	29- / 53+^*^	18- / 32+^*^	<0.001
Mean tau-PET follow-up, years^b^	1.72 (0.53)	1.64 (0.57)	1.45 (0.71)	1.37 (0.49)	0.299
**BioFINDER-1**					
	**CN Ab-/Tau-** **(*n*=36)**	**CN Ab+** **(*n*=30)**	**MCI Ab+** **(*n*=26)**	**AD dementia** **(*n*=46)**	***p*-value**
Age, years	73.86 (7.27)	73.80 (7.44)	71.73 (9.72)	71.43 (7.34)	0.274
Sex, M/F	19 / 17	12 / 18	18 / 8	25 / 21	0.187
Education, years	12.42 (3.80)	11.93 (3.72)	12.21 (3.93)	12.22 (3.75)	0.928
MMSE	28.86 (1.10)^‡,§^	28.83 (1.15)^‡,§^	25.58 (3.06)^*,†,§^	20.96 (4.87)^*,†,‡^	< 0.001
APOE e4 carriers -/+	29- / 7+^†,‡,§^	8- / 22+^*^	6- / 20+^*^	19- / 27+^*^	< 0.001
Mean tau-PET follow-up, years^c^	2.03 (0.47)	1.94 (0.32)	1.82 (0.12)	1.87 (0.34)	0.470

Abbreviations: *Aβ* Amyloid-beta, *AD* Alzheimer’s disease, *APOE* Apolipoprotein E, *CN* Cognitively normal, *F* Female, *M* Male, *MCI* Mild cognitive impairment, *MMSE* Mini-Mental State Exam

^a^available for 187 CN Aβ-/Tau-, 114 CN Aβ+, 82 MCI Aβ+, and 50 AD dementia

^b^subsample of 35 CN Aβ-/Tau-, 60 CN Aβ+, 39 MCI Aβ+, and 24 AD dementia

^c^subsample of 16 CN Aβ-/Tau-, 14 CN Aβ+, 7 MCI Aβ+, and 18 AD dementia

^*^Significantly different from CN Aβ-/Tau-

^†^Significantly different from CN Aβ+

^‡^Significantly different from MCI Aβ+

^§^Significantly different from AD dementia (significant after applying a Bonferroni-corrected α-threshold of 0.0125)

### Higher cortical myelin is associated with lower tau-PET

**Fig. 2:**
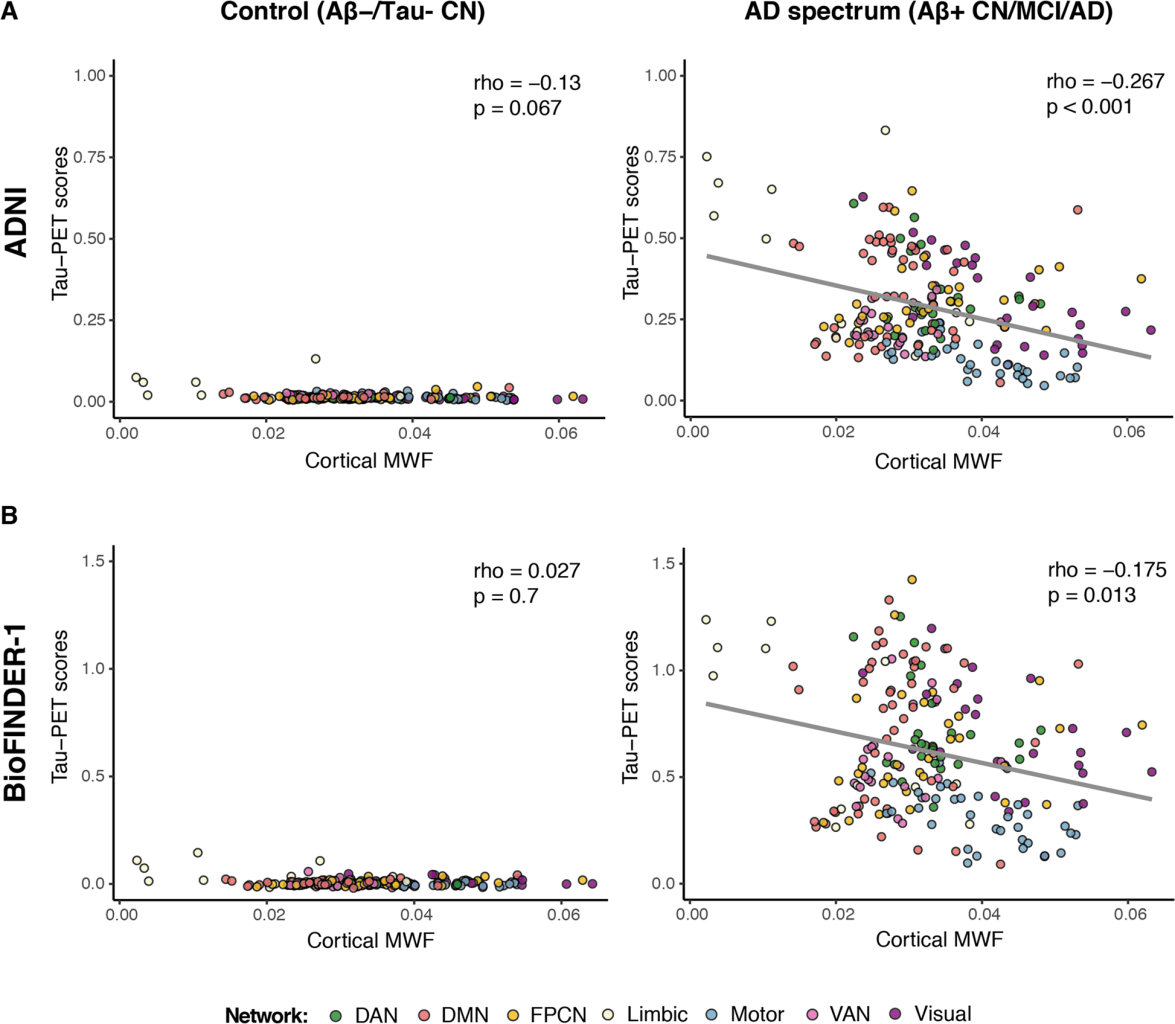
Association between cortical MWF and baseline tau-PET scores. Scatterplots showing the association between ROI levels of MWF and tau-PET scores for controls (CN Aβ − /Tau − ; left column) and AD spectrum (Aβ + participants; right column) from the ADNI (**A**) and BioFINDER-1 (**B**) cohorts. The coloring indicates for each ROI the major functional network it belongs to. DAN, dorsal attention network; DMN, default-mode network; PFCN, fronto-parietal control network; VAN, ventral attention network; MWF, myelin water fraction

Our first aim was to test in a cross-sectional analysis whether higher myelin levels in gray matter regions were associated with lower tau-PET uptake in spatially corresponding regions. To this end, we first applied a 200 ROIs cortical brain-parcellation atlas (Fig. 1A) to a spatially normalized MRI-based MWF template from cognitively normal healthy adults (*n* = 50, mean age 25 years) [27] in order to extract normative MWF ROI values from cortical brain regions (Fig. 1B). Next, we obtained subject-level tau-PET SUVRs from the same 200 ROI based on the tau-PET scans in the ADNI and BioFINDER-1 cohorts (Fig. 1C). The tau-PET ROI SUVRs were weighted by tau-positivity probability [7, 12] in order to reduce the influence of off-target background binding (henceforth called tau-PET scores, see Supplementary Fig. 1). We used spatial correlation to assess whether those gray matter ROIs with higher MWF levels show lower tau-PET scores in AD (Fig. 1D). As hypothesized, we found that higher ROI-MWF values were associated with lower tau-PET scores in corresponding gray matter ROIs for Aβ + participants in both ADNI (rho =  − 0.267, *p* < 0.001; Fig. 2A) and BioFINDER-1 (rho =  − 0.175, *p* = 0.013; Fig. 2B). The analysis in the CN Aβ-/Tau- participants was not significant both in ADNI (rho =  − 0.13, *p* = 0.067; Fig. 2A) and BioFINDER-1 (rho = 0.027, *p* = 0.7; Fig. 2B), suggesting that the association between MWF and tau-PET scores is robust only in the group with biomarker evidence of AD.

In a sensitivity analysis, we analyzed whether the associations differed between groups stratified by APOE ε4 status. In a stratified analysis, the 95% CIs of the bootstrapped rho values of the association between ROI-MWF values and tau-PET scores overlapped between the APOE-ε4 carriers and APOE-ε4 non-carriers both in ADNI (APOE-ε4 carriers: CI [− 0.375, − 0.218], APOE-ε4 non-carriers: CI [− 0.318, − 0.05]) and in BioFINDER-1 (APOE-ε4 carriers: CI [− 0.187, − 0.019], APOE-ε4 non-carriers: CI [− 0.286, − 0.14]), indicating that there is no significant difference between the groups.

Furthermore, in an additional sensitivity analysis, we used tau-PET SUVRs instead of tau-PET scores. We found that higher ROI-MWF values were associated with lower tau-PET SUVRs in corresponding gray matter ROIs for Aβ + participants in both ADNI (rho =  − 0.348, *p* < 0.001; Supplementary Fig. 2A) and BioFINDER-1 (rho =  − 0.255, *p* < 0.001; Supplementary Fig. 2B).

### Higher myelination of fiber tracts attenuates rates of longitudinal tau-PET increase in connected regions

**Fig. 3:**
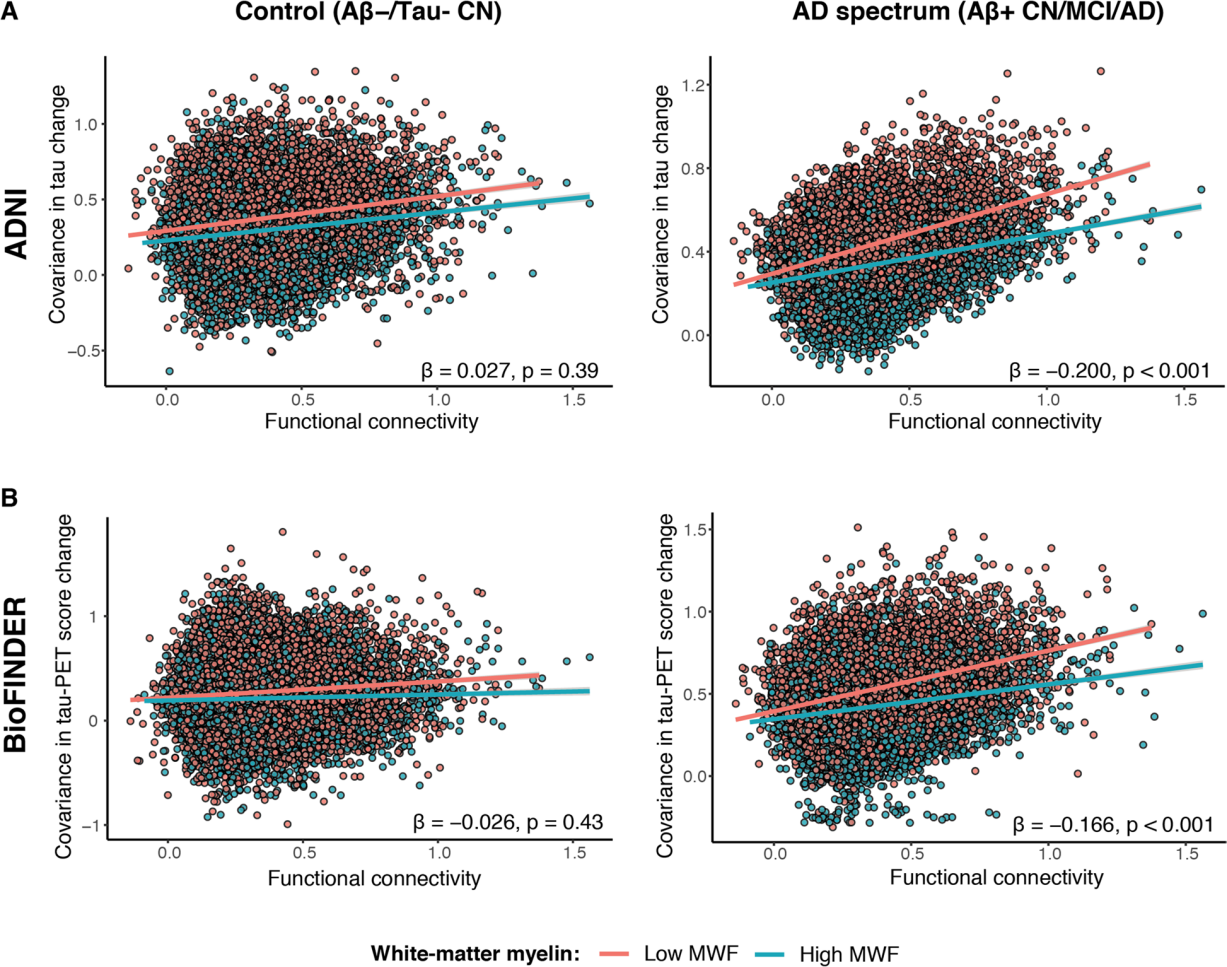
Interaction between functional connectivity and MWF in fiber-tracts on covariance in a tau-PET score change. Regression plots illustrating covariance in tau-PET change as a function of both functional connectivity and MWF in fiber-tracts (binarized by median spit) in the controls (CN Aβ − /Tau − ; left column) and AD spectrum (Aβ + participants; right column) groups of the ADNI (**A**) and BioFINDER-1 (**B**) cohorts. Red line is the regression line for participants with values < median MWF, and the blue regression line is for participants with values > median MWF. For the statistical analyses, MWF was used as a continuous measure and was stratified to high and low only for illustrational purposes. MWF, myelin water fraction

We and others previously reported that tau-PET preferentially progresses between closely connected brain regions [9]. Here, we tested whether myelination of fiber tracts modulates the rate of connectivity-dependent tau-PET accumulation in Aβ + participants. We addressed this hypothesis in a subset of Aβ + participants who had longitudinal tau-PET (ADNI: *n* = 123, mean FU interval = 1.53 [0.69–3.95] years; BioFINDER-1: *n* = 39, mean FU interval = 1.87 [1.21–2.78] years). Adopting our previously established approach to assess connectivity-based tau accumulation [9, 29], we first estimated the functional connectivity between each of the ROIs (Fig. 1G). Next, in order to determine whether functionally connected regions show similar rates of tau accumulation, we computed the covariance in tau-PET score change (i.e., the level of similarity in tau change between two regions; Fig. 1E, F). We then determined the level of myelin in the fiber tracts connecting each pair of ROIs to test whether myelin levels modulate the association between the functional connectivity and the change in tau-PET in the connected ROIs. To this end, we obtained the normative fiber-tract myelin levels from the same MWF template as used before, this time masked by an ROI-to-ROI structural connectivity matrix based on diffusion MRI data from 100 healthy individuals assessed in the HCP (Fig. 1H, I). In a linear regression analysis, we tested the interaction functional connectivity by MWF in fiber-tracts as predictors of the covariance in tau change matrix (Fig. 1J). We found a significant interaction of functional connectivity by fiber-tract MWF on covariance in tau change in both ADNI (*β* =  − 0.185, *p* < 0.001; Fig. 3A) and BioFINDER-1 (*β* =  − 0.166, *p* < 0.001; Fig. 3B), where regions that were connected by higher myelinated fiber tracts showed a lower association between functional connectivity and the rate of change in tau-PET scores. Results remained consistent when controlling the assessment of covariance in tau change for age, sex, education, APOE ε4 status, diagnosis, and site (ADNI: *β* =  − 0.114, *p* < 0.001; BioFINDER-1: *β* =  − 0.066, *p* = 0.029). These associations were not significant in the control groups (ADNI: *β* = 0.027, *p* = 0.39; BioFINDER-1: *β* =  − 0.026, *p* = 0.43; Fig. 3A, B). These results suggest that in the groups with biomarker evidence of AD, myelin is associated with an attenuated rate of connectivity-dependent tau-PET increase over time.

### The role of amyloid deposition in the associations between tau and MWF

In sensitivity analyses, we tested whether the above-mentioned associations were driven by regional amyloid pathology. Amyloid-PET was acquired in close temporal proximity to baseline tau-PET in 168 Aβ + participants in ADNI (mean interval = 20.5 [1–156] days) and in all participants in BioFINDER-1 (cross-sectional amyloid PET only).

First, we tested the association between cortical MWF and tau-PET scores, controlling for regional amyloid-PET SUVRs. We found that higher cortical MWF was associated with lower tau-PET scores in corresponding gray matter ROIs independent of amyloid-PET in ADNI (rho = -0.21, *p* = 0.003), but the association no longer reached significance in BioFINDER-1, although exploratory analysis showed that the association between cortical MWF and tau-PET was consistently independent of amyloid-PET in the non-demented group (CN Aβ + and MCI Aβ +) in both ADNI (rho =  − 0.241, *p* = 0.0006) and BioFINDER-1 (rho =  − 0.141, *p* = 0.046). These results suggest at the descriptive level, that especially in the early stage of AD, when tau-PET has not yet globally spread, the spatial association between myelin and tau deposition in the gray matter is independent of Aβ.

Next, we tested whether the association between higher fiber-tract MWF and lower connectivity-dependent longitudinal tau-PET accumulation remained significant in ADNI (no longitudinal amyloid-PET was obtained in BioFINDER-1). When controlling for the covariance in amyloid-PET change, the interaction functional connectivity by MWF in fiber tracts on covariance in tau change remained significant (*β* =  − 0.07, *p* = 0.005), suggesting that the association between higher levels of myelin and lower tau-PET accumulation was not dependent on differences in Aβ.

## Discussion

We combined for the first time high-resolution MRI-assessed mapping of myelin, resting-state fMRI functional connectomics, and subject-level tau-PET imaging in two independent samples of deeply characterized elderly individuals with biomarker evidence of AD. In the current study, we found a close spatial association between higher myelin levels and lower cross-sectional and longitudinal increase in tau-PET accumulation. Specifically, our first major finding showed that higher levels of myelin as assessed by MWF were consistently associated with lower tau-PET uptake in corresponding gray matter regions. This result suggests that brain regions with higher myelin levels are less prone to accumulate fibrillar tau. Our second major finding concerned the longitudinal progression of tau-PET between connected brain areas. We revealed that higher MWF levels in fiber tracts were associated with lower connectivity-dependent rates of tau-PET increases. This result suggests that higher fiber-tract myelination is associated with attenuated spreading of fibrillar tau in the brain. Although we caution against a causative interpretation, our current findings suggest that higher regional levels of myelin are associated with higher resistance against the susceptibility and rate of progression of fibrillar tau.

Our findings significantly advance previous qualitative brain autopsy studies that suggested an association between myelin and tau pathology [21, 26, 41], but were limited to qualitative visual inspection of sparsely sampled post-mortem data of tau pathology and myelin maps [26]. Here, using a longitudinal tau-PET approach, we show that higher fiber-tract myelination is associated with a lower connectivity-dependent rate of tau-PET progression. We thus advance previous findings on the association between the connectivity-based prediction of faster tau-PET accumulation in regions which are closely connected [9, 12, 42, 43], demonstrating that such connectivity-related increases in tau-PET accumulation are attenuated for regions connected by higher myelinated fiber-tracts. Together, our findings strongly support the notion that higher myelination is associated with lower susceptibility to tau accumulation and contribute to explain why some regions are highly vulnerable to tau pathology whereas others are relatively spared.

The mechanisms that may underlie a protective effect of higher myelin on lower tau pathology are poorly understood. A possible explanation is that lower myelinated axons are more prone to myelin damage, where subsequent remyelination processes entail higher phosphorylation of tau and subsequent formation of fibrillar tau [44, 45]. Myelin damage has been observed previously in histochemical and neuroimaging studies in AD [46–49], and recent single-cell studies provide converging results showing that alterations in oligodendrocytes, a cell type involved in myelination, are prominent in AD [50, 51]. Experimentally induced myelin damage leads to the activation of tau-targeting kinases entailing hyperphosphorylation of tau [22, 52], and could trigger further spreading of tau pathology [53, 54]. At the molecular level, the Fyn kinase, which is a key signaling molecule that binds tau in axonal microtubules [55], becomes activated during remyelination efforts triggered by myelin disturbances [56]. In mouse models of tauopathies, blocking Fyn kinase completely prevented the formation of neurofibrillary tangles [57]. Together, these results suggest the possibility that myelin-triggered Fyn kinase may play a role in the formation of tau tangles in AD. Alternatively, myelin alterations may enhance the development of tau pathology via increasing microglia activity. Previous research showed that myelin changes are associated with increased disease-associated microglia (DAM) gene-expression signatures triggering microglial uptake of myelin lipids [24]. The uptake of lipid droplets from myelin debris may render microglia overburdened and senescent [23, 58]. Furthermore, myelin-related microglia activation increases the inflammasome [23], which facilitates the development of fibrillar tau pathology [59]. Therefore, myelin alterations that occur preferentially in lower myelinated brain regions (for review see [60]) can trigger a type of microglia activation that entails a suboptimal response to developing tau pathology and thus indirectly enhances tau pathology.

Myelin damage may also lead to enhanced development of amyloid plaques [21]. Recent studies in transgenic mouse models of amyloid pathology suggest that acute demyelination may occur *upstream* of amyloid deposition by interfering with a TREM2-related microglia activation signature, thus leading to enhanced amyloid deposition [25]. Thus, more vulnerable, thinner myelinated regions may engage in dysfunctional microglial activity resulting in less efficient removal of AD pathology. However, our current results do not suggest that regional differences in the level of amyloid-PET explain the association between brain regions connected by lower myelinated fiber tracts and regional tau-PET accumulation. In the larger ADNI sample, the association between MWF in gray matter or fiber tracts with tau-PET remained significant after controlling for amyloid-PET, although in BioFINDER-1 there was a nominal association of cortical MWF with tau-PET only in non-demented participants. These results suggest that any association between myelin and Aβ deposition unlikely accounts for the association with tau-PET.

In summary, regions with higher myelination may be less vulnerable to late-life myelin alterations and thus a pathological cascade that includes the development of AD pathologies.

Our findings have important clinical implications. First, our results suggest that myelin adds to the connectivity-based prediction of regional increases in tau-PET [9, 12], and may thus contribute to patient-tailored outcome parameters on treatment effects on tau-PET changes [61]. Second, the current findings suggest myelin to be a potential target for the prevention and treatment of AD [62]. Myelination is a druggable target [63, 64], and thus, pharmacological stimulation of myelination is a putative therapeutic target in AD. Clemastine, a licensed H1 histamine, has been recently shown to enhance the differentiation of oligodendrocyte precursor cells and myelination [65, 66], where clinical trials repurposing clemastine for the treatment of multiple sclerosis have shown improvement of symptoms [67]. Treatment of transgenic mouse model of Aβ with clemastine showed alleviated oligodendrocyte progenitor cell and myelin loss, reduced Aβ deposition and ameliorated memory loss [45, 68]. Together, these results suggest that myelin is a putative drug target in AD and encourage future studies to test the effect of myelin treatment on tau pathology.

### Limitations

Several caveats should be considered when interpreting our results. First, current MRI acquisition sequences are imperfect measures of myelin and are susceptible to the iron content in the brain tissue [69]. However, we employed an MR-based myelin water imaging [27], which has been extensively validated by histopathology [70, 71] and shows one of the strongest associations with post-mortem assessed myelin levels compared to alternative MRI measures of myelin [72]. Also, we avoided a potential confounding of the MRI myelin signal with disease-related iron and white matter alterations by using a myelin water imaging template that was obtained in healthy individuals [27]. Therefore, the current findings are unlikely to be driven by spurious associations with off-target MRI signals. Second, in the current study, we used a myelin template from healthy individuals (*n* = 50, mean age 25 years) and did not assess myelin changes in the AD patients. While the use of a template reflects more accurately pre-morbid inter-regional differences in myelination, demyelination has been observed in aging [73], small vessel disease [74], and AD [75] in association with amyloid plaques [48] and fibrillar tau [46]. Hence, myelin changes may alter the rate of change in tau-PET. Patient-specific levels of myelin were not available in the current study, but the hypothesis is that regions with lower levels of myelin are more prone to undergo demyelination which then triggers the development of tau pathology.

## Conclusions

Our findings show for the first time that higher myelination is associated with slower tau progression in AD. Our results demonstrate that myelin maps inform the connectome-based prediction of pathology-spreading models, and thus also pave the way for future clinical studies in which the individual interregional differences in myelination can be assessed for patient-tailored prediction of disease progression.

## Supplementary Information

**Additional file 1: Supplementary figure 1.** Brain renderings of cortical myelin within the MWF template and tau-PET scores among controls and AD participants. **Supplementary figure 2.** Association between cortical MWF and baseline tau-PET SUVRs.

## Data Availability

The data used in this study were obtained from the Alzheimer’s disease Neuroimaging Initiative and are available from the ADNI database (adni.loni.usc.edu) upon registration and compliance with the data usage agreement. Data from the BioFINDER-1 cohort are available from the authors upon request. Resting-state and diffusion-weighted data of the HCP cohort are freely available online (https://db.humanconnectome.org). MWF template is freely available online (https://sourceforge.net/projects/myelin-water-atlas/).

## Abbreviations

AD: Alzheimer’s disease
ADNI: Alzheimer’s Disease Neuroimaging Initiative
ANTs: Advanced normalization tools
APOE: Apolipoprotein E
Aβ: Beta-amyloid
CDR: Clinical Dementia Rating
CN: Cognitively normal
DWI: Diffusion-weighted imaging
FU: Follow-up
HCP: Human connectome project
MCI: Mild cognitive impairment
MNI: Montreal Neurological Institute
MMSE: Mini-Mental State Examination
MRI: Magnetic resonance imaging
MWF: Myelin water fraction
PET: Positron emission tomography
ROI: Region of interest
SUVR: Standard uptake value ratio
